# Supplementary material for: FERN – a Java framework for stochastic simulation and evaluation of reaction networks
Source: BMC Bioinformatics. 2008 Aug 29;9:356. doi: 10.1186/1471-2105-9-356 (PMC2553347; doi:10.1186/1471-2105-9-356)
Supplement: Additional file 1 — FERN distribution, Version 1.3. This archive contains the FERN source code and binaries as well as documentation and example models in FernML and SBML. [file 1471-2105-9-356-S1.zip › fern/doc/javadoc/fern/tools/functions/Probability.html]

Probability


---


|  |  |  |
| --- | --- | --- |
| **Overview** \| **Package** \| **Class** \| **Use** \| **Tree** \| **Deprecated** \| **Index** \| **Help** | |  |
| PREV CLASS   **NEXT CLASS** | **FRAMES**    **NO FRAMES**     **All Classes** |  |
| SUMMARY: NESTED \| FIELD \| CONSTR \| METHOD | DETAIL: FIELD \| CONSTR \| METHOD |  |


---


## fern.tools.functions Interface Probability

**All Known Implementing Classes:**: Probability.Constant, Probability.ReactionProbability

---

``` public interface Probability ```

Function delegate for conditional probabilities.

**Author:**
:   Florian Erhard

---

| **Nested Class Summary** | |
| --- | --- |
| `static class` | `Probability.Constant`             Implementation that yields a constant (and hence independent) probability. |
| `static class` | `Probability.ReactionProbability`             Implementation for the reaction probability for the autocatalytic network creation. |


| **Method Summary** | |
| --- | --- |
| `double` | `getProb(int l1, int l2)`             Gets the probability depending on two integers. |

**Method Detail**

### getProb

```
double getProb(int l1,
               int l2)
```

:   Gets the probability depending on two integers.

    :   **Parameters:**: `l1` - interger 1: `l2` - integer 2 **Returns:**: probability


---


|  |  |  |
| --- | --- | --- |
| **Overview** \| **Package** \| **Class** \| **Use** \| **Tree** \| **Deprecated** \| **Index** \| **Help** | |  |
| PREV CLASS   **NEXT CLASS** | **FRAMES**    **NO FRAMES**     **All Classes** |  |
| SUMMARY: NESTED \| FIELD \| CONSTR \| METHOD | DETAIL: FIELD \| CONSTR \| METHOD |  |


---
